# What web-based intervention for chronic cancer-related fatigue works best for whom? Explorative moderation analyses of a randomized controlled trial

**DOI:** 10.1007/s00520-022-07223-y

**Published:** 2022-06-21

**Authors:** Melanie P. J. Schellekens, Fieke Z. Bruggeman-Everts, Marije D. J. Wolvers, Miriam M. R. Vollenbroek-Hutten, Marije L. van der Lee

**Affiliations:** 1 Scientific Research Department, Helen Dowling Institute, Centre for Psycho-Oncology, Bilthoven, The Netherlands; 2 Department of Medical and Clinical Psychology, Tilburg University School of Social and Behavioral Sciences, Tilburg, The Netherlands; 3 Telemedicine Group, Faculty of Electrical Engineering, Mathematics, and Computer Science, University of Twente, Enschede, The Netherlands; 4 ZGT Academy, ZiekenhuisGroep Twente, Almelo, The Netherlands

**Keywords:** Chronic cancer-related fatigue, Fatigue, Cancer survivors, Mindfulness-based cognitive therapy, Activity intervention, Moderation analysis

## Abstract

**Purpose:**

Approximately 25% of cancer patients suffer from chronic cancer-related fatigue (CCRF), which is a complex, multifactorial condition. While there are evidence-based interventions, it remains unclear what treatment works best for the individual patient. This study explored whether baseline characteristics moderated the effect of web-based mindfulness-based cognitive therapy (eMBCT) versus ambulant activity feedback (AAF) and a psycho-education control group (PE) on fatigue in patients suffering from CCRF.

**Methods:**

In a randomized controlled trial, participant suffering from CCRF participated in either eMBCT, AAF, or PE. Complete data of the treatment-adherent sample (≥ 6 sessions) was used to explore whether sociodemographic, clinical, and psychological characteristics at baseline moderated the intervention effect on fatigue severity at 6 months.

**Results:**

A trend showed that baseline fatigue severity and fatigue catastrophizing moderated the intervention effect. That is, at low levels of fatigue severity and catastrophizing, patients benefited more from AAF than from eMBCT and at high levels of fatigue severity and catastrophizing, patients benefited more from eMBCT than from PE.

**Conclusions:**

This study found some preliminary evidence on what treatment works best for the individual suffering from CCRF. These findings emphasize the potential gain in effectiveness of personalizing treatment. An alternative approach that might help us further in answering the question “what treatment works best for whom?” is discussed.

## Introduction

Fatigue is one of the most prevalent and disrupting side effects of cancer and its treatment. This fatigue differs from typical tiredness as it is not alleviated by rest or sleep, nor is it proportional to recent exertion [1]. In approximately 25% of patients, it persists for months to years after completion of cancer treatment [1]. This persisting fatigue is defined as chronic cancer‐related fatigue (CCRF). CCRF interferes with patients’ ability to work, their daily life activities, and social relationships and is often accompanied by distress [1, 2].

Evidence suggests CCRF is a complex multifactorial condition, affected by a range of physiological (e.g., inflammation), clinical (e.g., comorbidities, cancer treatment), and psychosocial factors (e.g., distress) [1]. Following the multifactorial etiology of CCRF, several types of treatments have proven effective in helping patients cope with CCRF. These include both psychosocial interventions and physical activity interventions [3]. So far, it remains unknown what intervention is most suitable for which patient. In order to provide patients with the best and most efficient care, we need to answer the question “What works best for whom?” Therefore, characteristics that influence the direction or magnitude of the effect of such interventions on cancer-related fatigue need to be identified.

Recently, a three-armed randomized controlled trial compared the effects of two online interventions (a psychologist-guided web-based MBCT (eMBCT) and a physiotherapist-guided ambulant activity feedback (AAF)) to an unguided active control group receiving psycho-education (PE) in severely fatigued cancer patients [4]. Results showed that both eMBCT and AAF were superior to PE in reducing fatigue [4]. This three-armed design provides an unprecedented opportunity to conduct moderation analyses and study for which subgroup of patients what type of intervention (i.e., eMBCT, AAF, or PE) is most beneficial. The aim of the current study is to explore the moderator effects of sociodemographic, clinical, and psychological characteristics on the effect of eMBCT, AAF, and PE on fatigue in patients suffering from CCRF.

## Methods

This moderation study was embedded in a three-armed randomized controlled trial [5]. The study was approved by the Twente Medical Ethical Committee (P12-26).

### Participants and procedure

Cancer survivors were recruited via various channels (e.g., patient organizations, social media, newspapers, health care professionals). Patients were invited to follow a web-based intervention for their fatigue but did not receive the exact content of the interventions in the advertisements. Inclusion criteria were having finished curative-intent cancer treatment at least 3 months previously after any type of cancer diagnosis, suffering from severe fatigue ever since (≥ 35 on the Checklist Individual Strength—Fatigue Severity [CIS-FS] subscale [4]), being ≥ 18 years old at disease onset, no current or former severe psychiatric comorbidity (e.g., suicidal ideation, psychosis), no current substance abuse, no cancer recurrence during study participation, and no dependence on wheelchair for daily activities.

After providing written informed consent, eligible patients were randomized to eMBCT, AAF, or PE [4]. Participants filled out questionnaires prior randomization (baseline, T0), 1 week after the 9-week intervention period (T1), and at follow-up (6 months, T2).

### Interventions

Interventions were similar in duration (9 weeks) and psycho-education content which participants received weekly via a website (eMBCT and AAF) or e-mail (PE). The psycho-education content came from the eMBCT program [7] and included information on fatigue, sleep hygiene, balancing energy during the day, and coping with worrying thoughts. Due to the similarities in duration and psycho-education content, treatment adherence was set at ≥ 6 sessions for all three interventions.

### eMBCT

The eMBCT-program is a 9-week web-based psychologist-guided intervention, based on the original MBCT protocol [6] and tailored to patients suffering from CCRF by including cancer- and fatigue-related psycho-education, and adapted movement exercises [7]. The treatment material consisted of nine modules, which the patient could consult by logging on to a password-secured website. Each module involved information about mindfulness, psycho-education on fatigue, and audio-guided mindfulness exercises (body scans, sitting meditations, gentle yoga exercises, and walking meditations). Patients were encouraged to practice and fill out their experiences in a diary on a daily basis. On an agreed-upon day of the week, the therapist replied to these diary entries, thereby guiding the patient through the program.

#### AAF

AAF is a 9-week web-based physiotherapist-guided protocolled intervention [8] using an activity coaching system that consists of the patient’s smartphone and an accelerometer. Patients set personal activity goals together with the therapist (i.e., (1) activate: becoming more active; (2) temper: taking rest in time; (3) balance: balancing activity and rest throughout the day and especially conserve energy in the morning). The coaching system supports patients in meeting these goals by showing real-time feedback about the accumulated activity relative to a personalized line of reference. Participants received tailored messages on their smartphone in order to increase, decrease, or balance their daily activities in ways that improve their energy levels. The feedback was not coupled to exercise training but to activities that can be easily performed in and around the house or office. For example, depending on the current deviation from the reference line, activating feedback messages included proposed behaviors such as “a nice stroll” or “a brisk walk” while tempering feedback messages included proposed behaviors such as “reading the newspaper.” Via a password-secured website, patients received weekly psycho-education on fatigue. On this website, participants described their experiences in a diary. On an agreed-upon day of the week, the therapist replied to these diary entries, thereby guiding the patient through the program.

#### PE

PE involved reading weekly non-reply emails for a period of 9 weeks, including the psycho-education on fatigue. In contrast to eMBCT and AAF, adherence was not actively monitored in PE. Only when participants informed the researcher they wanted to drop out before the sixth session, they were considered non-adherent.

### Measures

#### Outcome measure

*Fatigue severity* was assessed with the 8-item CIS-FS [9]. The CIS has been validated, showed good psychometric properties, and has often been used with cancer survivors [6].

### Moderators

Sociodemographic and clinical moderators were assessed via self-report at T0: *gender*, *age*, *education level*, *cancer type*, and *fatigue duration*. Baseline *fatigue severity* was assessed with the CIS-FS [10]. *Fatigue catastrophizing* was assessed with the Fatigue Catastrophizing Scale (FCS) [6], which showed good psychometric properties and has previously been used in cancer survivors [11]. *Sense of control over fatigue* (confidence about one’s capacity to change fatigue) was measured with the self-efficacy scale (SE28), specifically adapted for people suffering from chronic fatigue [12]. *Perceived activity* and *concentration* were assessed with the 3-item activity subscale and the 5-item concentration subscale of the CIS [12]. *Acceptance* (non-reactive attitude to inner experiences) and *presence* (focus on current experiences) were assessed with the 8-item acceptance and 6-item presence subscale of the Freiburg Mindfulness Inventory (FMI) [6], which has been validated in cancer survivors, showing good psychometric properties [13]. The 12-item Multidimensional Scale of Perceived Social Support (MSPSS) assessed *social support*, which shows good psychometric properties [13, 14].

### Statistical analysis

Analyses were based on complete cases of the treatment-adherent sample (participation in ≥ 6 sessions). Using the SPSS macro PROCESS [15], we evaluated a set of linear regression models to examine whether the effect of treatment (eMBCT vs. AAF vs. PE) on the outcome at T2 (fatigue severity) was moderated by the moderators at T0. Following recommendations by Hayes [16], we used the T0 measure of the outcome as a covariate to correct for individual differences at baseline. The multi-categorical independent variable for treatment condition was dummy-coded, with “0” indicating absence and “1” indicating presence of each treatment condition. eMBCT served as the reference condition. Both dummy variables representing the independent variable of treatment, the moderator, and the two resulting interaction terms between each dummy variable and the moderator were included in the analyses. A significant increase in explained variance (Δ*R*^2^) resulting from adding the product terms to the model already containing the dummy variables and the main effect of the moderator was considered evidence for moderation [16].

Simple slope analysis was used to interpret the significant moderation effects [17]. PROCESS for SPSS provides omnibus tests, indicating if there is a difference in estimated outcome between the conditions at three different values of the moderator: 1 SD below the mean (“low”), the mean (“moderate”), and 1 SD above the mean (“high”). When the omnibus test for a specific level of the moderator was significant, pairwise inferences explored if eMBCT differed from AAF or PE at that level of the moderator.

## Results

### Study sample

**Fig. 1:**
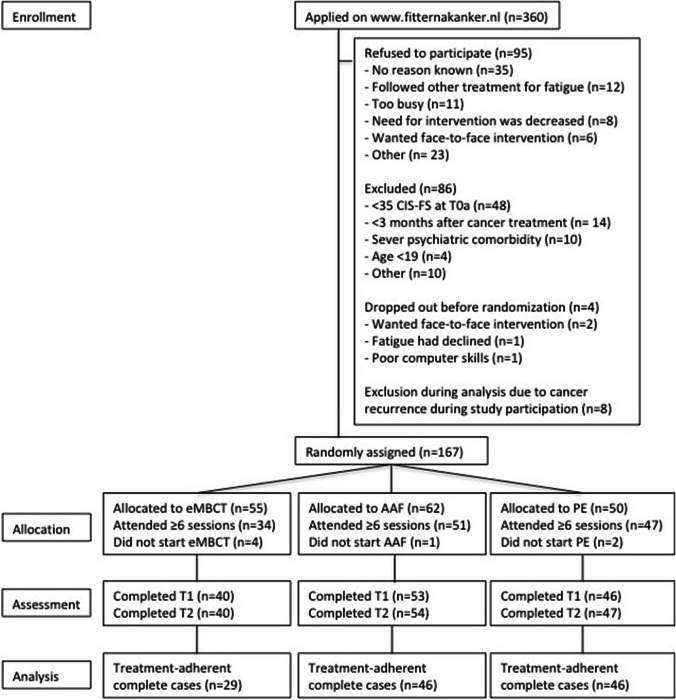
Flowchart of participants

Of the 360 people who applied on the website between March 2013 and June 2015, 95 (26%) refused to participate, 86 (24%) were excluded, 4 (1%) dropped out additionally before randomization, and 8 (2%) were excluded prior analysis due to cancer recurrence during study participation, resulting in 167 participants. See Fig. 1 for participant flowchart. Due to a temporary error in the website’s randomization algorithm, participants were not allocated 1:1:1 for a period of 6 months, resulting in unequal sample sizes for the conditions: eMBCT (*n* = 55), AAF (*n* = 62), and PE (*n* = 50). Of those 167 participants, 132 (79%) were considered treatment-adherent (participated in ≥ 6 sessions). Reasons for dropping out the interventions included lack of confidence the intervention would be effective, no desire for treatment because fatigue had already reduced considerably during the first sessions, and preference for face-to-face contact. Specific reasons for dropping out of eMBCT included the high intensity of the program and difficulties using the eMBCT portal. Reasons for dropping out of AAF were mainly technical problems and poor usability of the accelerometer. Intervention dropout was higher in eMBCT (21/55; 38%) than in AAF (11/62; 18%) and PE (3/50; 6%), which is probably due to the high intensity of the eMBCT program, including daily mindfulness practice. Adherent patients did not differ from non-adherent patients regarding baseline characteristics.

Eventually, 121 participants (92%) of the treatment-adherent sample completed the T2 assessment: eMBCT (*n* = 29), AAF (*n* = 46), or PE (*n* = 46). Baseline characteristics of the complete cases of the treatment-adherent sample are shown in Table 1. No significant differences were found between interventions, with the exception that eMBCT participants were more severely fatigued at baseline than PE participants (*p* = 0.021).

**Table 1:** Baseline characteristics of treatment-adherent sample with complete data

		eMBCT (*n* = 29)	AAF (*n* = 46)	PE (*n* = 46)
Demographic characteristics, *n* (%)				
Gender	Female	18 (62.1)	33 (71.7)	37 (80.4)
	Male	11 (37.9)	13 (28.3)	9 (19.6)
Age, M (SD)		53.24 (11.13)	58.02 (9.57)	56.15 (8.66)
Education level^ab^	Low/middle	10 (34.5)	24 (52.2)	16 (34.8)
	High	19 (65.5)	21 (45.7)	30 (65.2)
Clinical characteristics, *n* (%)				
Type of cancer	Breast	12 (41.4)	18 (39.1)	26 (56.6)
	Other	17 (58.6)	28 (60.9)	20 (43.5)
Length of fatigue^a^	< 2 years	9 (31.0)	17 (37.0)	22 (47.8)
	> 2 years	20 (69.0)	28 (60.9)	24 (52.2)
Psychological characteristics, M (SD)				
Fatigue severity (CIS-FS)^c^		44.56 (6.79)	42.46 (6.60)	40.15 (9.35)
Fatigue catastrophizing (FCS)		21.99 (5.36)	22.05 (5.97)	21.40 (5.64)
Sense of control over fatigue (SE28)		17.52 (2.42)	18.15 (2.04)	18.04 (2.80)
Perceived activity (CIS-A)		10.38 (4.83)	10.33 (3.77)	10.98 (5.27)
Concentration (CIS-C)		16.52 (6.96)	18.59 (7.21)	19.24 (7.56)
Acceptance (FMI-A)		20.59 (3.73)	21.63 (3.97)	22.52 (4.81)
Presence (FMI-P)		16.79 (3.56)	17.59 (3.11)	18.30 (3.48)
Social support (MSPPS)		5.31 (1.18)	5.75 (0.94)	5.68 (1.24)

^a^In AAF, *n* = 1 is missing; ^b^low/middle = primary and secondary education; high = higher vocational training and university; ^c^eMBCT participants reported a higher level of fatigue severity than PE participants (*p* = 0.021)

### Moderation

Table 2 shows the results of the linear regression models to determine moderation of treatment effect. In all analyses, there was a significant main effect of intervention on fatigue severity at T2, indicating that eMBCT and AAF outperformed PE in reducing fatigue severity. None of the moderation effects resulted in a significant increase in explained variance of T2 fatigue severity. Baseline fatigue severity (*p* = 0.066) and fatigue catastrophizing (*p* = 0.084) did show a trend towards an overall moderation effect.

**Table 2:** Overall tests of moderation effects (the added value of the moderator x intervention interactions) on fatigue severity at T2. Moderation effects are corrected for the main effect of intervention, the main effect of the moderator, and baseline fatigue severity

	Full model			Overall test of moderation effect		
Moderator	*F* (df)	*p*	*R*^2^	*F* (df)	p	*R*^2^-change
Gender (ref: males)	6.59 (6, 114)	< 0.001	0.258	0.41 (2, 114)	0.665	0.005
Age	6.56 (6, 114)	< 0.001	0.257	0.31 (2, 114)	0.734	0.004
Education level (ref: low/middle)	6.86 (6, 113)	< 0.001	0.267	0.88 (2, 113)	0.417	0.011
Type of cancer (ref: other)	7.52 (6, 114)	< 0.001	0.283	2.15 (2, 114)	0.121	0.027
Length of fatigue (ref: < 2 years)	6.74 (6, 113)	< 0.001	0.264	0.41 (2, 113)	0.661	0.005
Fatigue severity at T0	9.24 (5, 115)	< 0.001	0.287	2.77 (2, 115)	0.066	0.035
Fatigue catastrophizing	7.55 (6, 114)	< 0.001	0.285	2.53 (2, 114)	0.084	0.032
Sense of control over fatigue	6.63 (6, 114)	< 0.001	0.259	0.05 (2, 114)	0.949	< 0.001
Perceived activity	6.41 (6, 114)	< 0.001	0.252	0.01 (2, 114)	0.988	< 0.001
Concentration	7.00 (6, 114)	< 0.001	0.269	1.17 (2, 114)	0.316	0.015
Acceptance	6.80 (6, 114)	< 0.001	0.264	0.59 (2, 114)	0.555	0.008
Presence	6.74 (6, 114)	< 0.001	0.262	0.59 (2, 114)	0.558	0.008
Social support	7.40 (6, 114)	< 0.001	0.280	2.17 (2, 114)	0.119	0.027

**Fig. 2:**
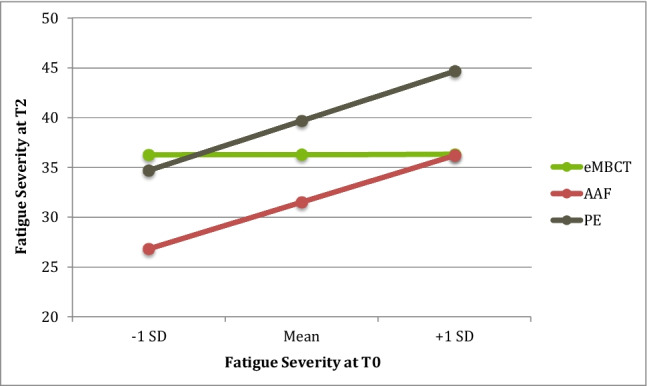
The effect of treatment on T2 fatigue severity at different levels (− 1 SD, Mean, + 1 SD) of the moderator baseline fatigue severity

Regarding baseline fatigue severity (Fig. 2), the omnibus tests showed that T2 fatigue severity differed between interventions for participants with low (F(2, 115) = 5.87, *p* = 0.004), moderate (F(2, 115) = 10.53, *p* < 0.001), and high (F(2, 115) = 6.70, *p* = 0.002) fatigue levels. Pairwise inferences showed that at low to moderate (CIS-FS = 34.16–42.09) baseline fatigue, eMBCT was outperformed by AAF (B =  − 9.44 (3.55), *p* = 0.009; B =  − 4.78 (2.09), *p* = 0.024, respectively); no differences between eMBCT and PE. At high (CIS-FS = 50.01) baseline fatigue, eMBCT outperformed PE (B = 8.35 (2.73), *p* = 0.003); no differences between eMBCT and AAF.

**Fig. 3:**
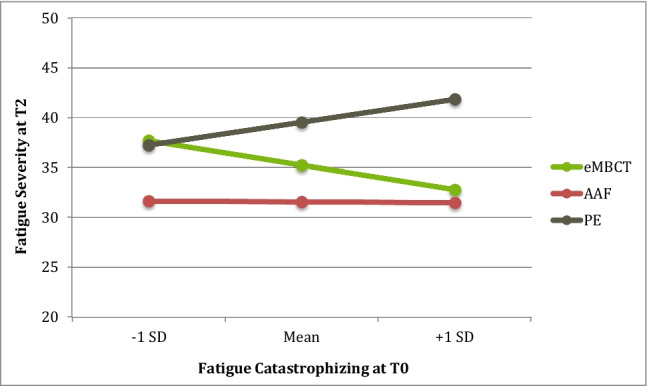
The effect of treatment on T2 fatigue severity at different levels (− 1 SD, Mean, + 1 SD) of the moderator fatigue catastrophizing

Regarding fatigue catastrophizing (Fig. 3), the omnibus tests showed that T2 fatigue severity differs between interventions at low (F(2, 114) = 3.22, *p* = 0.044), moderate (F(2, 114) = 9.88, *p* < 0.001), and high catastrophizing levels (F(2, 114) = 9.31, *p* < 0.001). Pairwise inferences showed that at low catastrophizing levels (FCS = 16.12), eMBCT was outperformed by AAF (B =  − 6.04 (2.98), *p* = 0.045); no differences were found between eMBCT and PE. At moderate to high (FCS = 21.79–27.46) catastrophizing levels, eMBCT outperformed PE (B = 4.30 (2.07), *p* = 0.040; B = 9.05 (2.95), *p* = 0.003, respectively); no differences were found between eMBCT and AAF.

## Discussion

The present study explored whether baseline sociodemographic, clinical, and psychological characteristics moderated the effect of eMBCT, AAF, and PE on 6-month fatigue severity. Some indication was found (marginally significant effect) that baseline fatigue severity and fatigue catastrophizing moderated the intervention effect on fatigue. None of the other characteristics moderated the intervention effect.

Regarding baseline fatigue severity, additional analyses suggested that in case of high fatigue, patients benefited more from eMBCT than from PE. These findings are in line with a recent individual patient data meta-analysis, showing that cancer patients with clinically relevant fatigue levels benefit more from psychosocial interventions than patients with non-clinical fatigue [17]. In the present study, all patients reported clinically relevant fatigue at baseline, indicating that in cases of “extreme” fatigue, eMBCT outperformed PE. Interestingly, when fatigue was less extreme but still clinically significant, AAF outperformed eMBCT, implying it seems more beneficial for patients to receive support on balancing activities in order to improve their energy levels, rather than train their awareness to help them cope with fatigue. Regarding fatigue catastrophizing, additional analyses suggested that at low catastrophizing levels, patients benefited more from AAF than from eMBCT, and at high catastrophizing levels, patients benefited more from eMBCT than from PE. Previous studies have shown that catastrophizing is an important predictor of fatigue severity and is linked to more daily life interference due to the fatigue [10]. Following these results, our findings suggest that when people catastrophize more, more personal support is warranted and psycho-education does not seem to offer enough support. A more intensive intervention, such as eMBCT or AAF, is required to help patients cope with fatigue. Similar to the moderating trend of baseline fatigue severity, patients benefit more from AAF than from eMBCT when patients catastrophize less. At these lower levels of catastrophzing, potentially less psychological factors might influence the CCRF experience and as such it can be more beneficial for patients to focus on balancing activities (AAF), rather than participate in a psychosocial intervention, such as eMBCT.

### Methodological issues

The design of this three-armed RCT in severely fatigued cancer patients provided a unique opportunity to study for which subgroup of patients what intervention is most beneficial. However, when interpreting the findings, the small sample size needs to be taken into account. The study was not powered to conduct moderation analysis and several patients did not adhere to the intervention, resulting in a small sample size. This raises the concern whether the negative findings are due to the study being underpowered rather than the effects being absent. This power issue also needs to be taken into account when interpreting the marginally significant moderation effects of baseline fatigue severity and fatigue catastrophizing. In light of these findings, it should also be noted that, probably due to the high intensity of the program, dropout appeared larger in eMBCT than in the other interventions, leading to a relatively small sample size in the eMBCT arm. Moreover, eMBCT patients reported higher fatigue levels at baseline than patients in AAF and PE. However, by including baseline fatigue in the moderation analysis, we controlled for this baseline difference between conditions. In addition, as with other psycho-oncology research, the majority of participants were middle-aged breast cancer patients. Moreover, the sample was mainly self-selected, resulting in a group of motivated participants. Although this is mostly in line with the characteristics of cancer patients seeking psychosocial support [18], this might limit generalizability to patients with other cancer types who are less motivated to participate in an online fatigue intervention.

### Person-based approach

The present moderation study provided limited insight into “what works best for whom?” A larger sample size could have helped us to better determine relevant moderators. However, a more person-centered approach might be more appropriate to answer this question. For example, a structured diary technique, such as the experience sampling method, in which participants receive questions multiple times a day for multiple days on end about their symptoms, thoughts, and feelings, allows closely monitoring of fatigue in patients’ daily living environment [19]. This results in an intensive longitudinal dataset, making it possible to examine the interactions between symptoms, cognitions, emotions, and behavior in a detailed, ecologically valid manner at the level of the individual patient [20]. The network approach offers a new way to gain insight into an individual’s symptom dynamics. It theorizes symptoms as elements of a complex dynamical system in which symptoms can trigger one another (e.g., sleep problems lead to fatigue and concentration problems, resulting in loss of enjoyment, which in turn can trigger a depressed mood) [20]. Such symptom networks can provide new insight into how one copes with fatigue, which could be helpful in determining what treatment would be most suitable for the individual patient [21].

## Conclusion

In sum, we found some preliminary evidence that baseline fatigue severity and fatigue catastrophizing moderated the effect of eMBCT versus AAF and PE on fatigue severity in CCRF patients. These findings emphasize the potential gain in effectiveness of personalized treatment. This work could help healthcare professionals to find the right treatment for their patients suffering from CCRF. However, more research is needed to substantiate and improve guidance of personalized treatment for CCRF. The network approach [22] might help us further in answering the question “what treatment works best for whom?”.

## Data Availability

The data that support the findings of this study are available from the corresponding author, MS, upon reasonable request.

## Code availability

N/A.
